# A Rare Complex BRAF Mutation Involving Codon V600 and K601 in Primary Cutaneous Melanoma: Case Report

**DOI:** 10.3389/fonc.2020.01056

**Published:** 2020-07-10

**Authors:** Francesca Consoli, Gianluca Barbieri, Matteo Picciolini, Daniela Medicina, Mattia Bugatti, Valeria Tovazzi, Barbara Liserre, Claudia Zambelli, Fausto Zorzi, Alfredo Berruti, Emanuele Giurisato, William Vermi

**Affiliations:** ^1^Unit of Medical Oncology, Spedali Civili di Brescia, Brescia, Italy; ^2^Diatech Pharmacogenetics SRL, Jesi, Italy; ^3^Unit of Anatomic Pathology, Spedali Civili di Brescia, Brescia, Italy; ^4^Department of Pathology, Fondazione Poliambulanza, Brescia, Italy; ^5^Department of Medical and Surgical Specialties, Radiological Sciences, and Public Health, University of Brescia, Brescia, Italy; ^6^Division of Cancer Sciences, School of Medical Sciences, Faculty of Biology, Medicine and Health, The University of Manchester, Manchester, United Kingdom; ^7^Department of Biotechnology Chemistry and Pharmacy, University of Siena, Siena, Italy; ^8^Department of Molecular and Translational Medicine, University of Brescia, Brescia, Italy; ^9^Department of Pathology and Immunology, Washington University School of Medicine, St. Louis, MO, United States

**Keywords:** metastatic melanoma, BRAF, mutation, NGS, BRAF inhibitors, MEK inhibitors

## Abstract

BRAF is one of the most common mutated kinases detected in human cancer, particularly in cases of primary cutaneous melanomas (PCM). Mutations of the BRAF proto-oncogene, at the p.V600 codon, has been detected in more than 50% of primary and metastatic melanoma cells in clinical samples. In addition to the most frequent BRAF p.V600E mutation, corresponding to the single base pair substitution c.1799T>A, rarer mutations, within and outside the V600 codon, have been described. Expectedly, BRAF and MEK inhibitors (or their combination) have been poorly explored as potential therapeutic strategies in metastatic melanomas harboring this rare mutation. By using a set of sequencing techniques and immunohistochemistry, this work reports the genomic and clinical features of two melanoma patients showing a rare complex mutation affecting codon V600 and K601 of the BRAF gene, leading to a V600E2; K601I change. Specifically, these two patients show a distinct clinical behavior and significantly differ in their responses to BRAF and MEK inhibitors. Indeed, although this treatment has proven to be effective and safe in both cases, the observed variability between the two patients resulted as a direct consequence of the baseline extent of brain involvement, intracranial treatment failure as well as on the PTEN status.

## Background

BRAF is one of the most common mutated kinases in human cancer (1). Forty-to-fifty percent of primary cutaneous melanomas (PCM) harbor a BRAF mutation (1, 2) located at the p.V600 codon, leading to constitutive activation of serine-threonine kinase activity, together with the corresponding downstream signal transduction in the MAPK (mitogen activating proteins kinase) pathway.

The most frequent BRAF hot-spot mutation in PCM targets the V600 amino acid residue, in the activation segment of the gene, encoded by a codon within the exon 15. It has been reported that eighty percent of mutated melanomas (MM) result in single amino-acid substitution of valine by glutamic acid, now referred to as the V600E mutation (1, 3). Substitution at codon V600 might also generates non-V600E changes, including V600D/E2/K and R, which can result in strong activation of BRAF kinase activity. In the BRAF p.V600 mutant, excessive activation results as a consequence of increased exposure of its activation segment to interactions with a small hydrophobic amino acid at the 600 (valine) which is substituted with a hydrophilic residue (glutamic acid). The catalytic activity remains insensitive to the regulatory process and independent of RAS activation (4, 5). As a consequence, oncogenic mutations favor cellular proliferation and reprogramme metabolism processes to sustain cellular growth (6). The identification of oncogenic BRAF mutations has led to the development and identification of highly selective BRAF inhibitors in the clinical setting (7, 8). Recently published phase III clinical trials have demonstrated the improved responsiveness of BRAFV600 melanoma patients using BRAF and MEK inhibitors (BRAFi and MEKi) in combination as compared to BRAFi alone and now forms part of a standardized therapeutic regimen approach in the clinical setting (9–11).

Rare variants of BRAF mutation, named non-V600 variants, have been documented in melanomas and occur in ~5–16% of clinical cases (3). BRAF non-V600 mutants have demonstrated different effects with regard to BRAF kinase activation pathways. These include two subgroups with different activation of MAPK pathway: class II mutations (e.g., K601, L597) or class III mutations (e.g., G466, N581, D594) (3). For each class of BRAF mutation, pre-clinical, and clinical studies have demonstrated distinct oncogenic mechanisms which in turn might predict different therapeutic strategies (3, 12, 13).

Among class II BRAF mutations, those affecting the K601 codon targets the activation segment of BRAF adjacent to the V600 position and this results in an increased activation of the MAPK pathway (3, 14). Among K601 variants, K601E occurs in about one percent of melanomas, resulting in a single amino acid substitution of lysine by glutamic acid. This variant was associated to an increase of intra-cellular phospho-MEK and ERK levels in preclinical models. Furthermore, recent *in vitro* studies, confirmed a reduction of phospho-ERK signaling in BRAF K601E mutated tumors, treated with a MEK inhibitor (15). Even though the vast majority of K601 mutations consist of a single nucleotide substitution (i.e., K601E, K601N, K601T), more complex mutations determining fusion proteins have been recognized. Additionally, the molecular characterization of BRAF mutations has been recently improved by the next generation sequencing (NGS), which provides more detailed genomic information when compared to some traditional sequencing methods (5, 16, 17). NGS allows the detection and characterization of complex genetic alterations of BRAF that could lead to the development of a more “patient-tailored” treatment option in the clinical setting (18).

In this study, the authors describe two cases of PCM, with the same complex BRAF mutation involving both V600 and K601 codons but showing a distinct clinical behavior and variable response to the combination of dabrafenib plus trametinib. Furthermore, the authors carried out an analysis of the new and existing clinical data “pooled” from several sources in order to explore the role of BRAF and MEK inhibitors in patients harboring tandem mutations (19).

## Method

Data analysis was detailed in Supplementary Material. A written informed consent was obtained from the patients, before commencement of any research studies.

## Case Presentation

### Clinical and Genetic Findings: Patient#1 (Pt#1)

**Figure 1:**
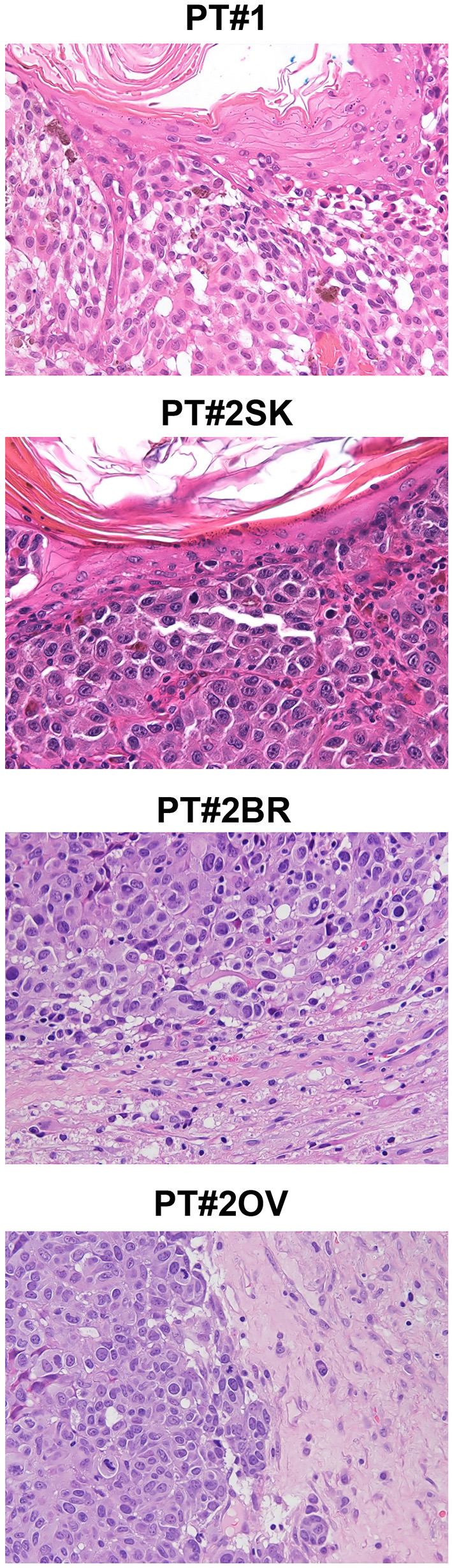
Melanoma histology in Patient 1 (PT#1) and Patient 2 (PT#2) at different sites. Sections of PT#1 and PT#2 melanomas are from different melanoma sites as indicated and stained for haematoxilin and eosin. Magnification: 200x; sk, skin; ov, ovary; br, brain.

Resection of a cutaneous melanoma of the trunk was performed in a 74-years old male (Breslow thickness of 4.9 mm, ulceration present, mitotic rate 14 mm^2^) (Figure 1). After sentinel lymph node dissection, he was staged as IIIB, according to AJCC 7th edition. After 4 years from initial diagnosis, he progressed in brain, lung, and lymph nodes, with normal LDH levels and performance status (PS) was ECOG 1, due to a mild dysarthria. The patient had no comorbidities, nor past interventions; no history for familial melanoma was reported. At the baseline, the sum of intra- and extracranial lesions diameters (SLD) was 92 mm. The largest brain metastasis, over a total of two lesions, had a diameter of 24 mm and involved the left parietal region. A biopsy of a mediastinal lymph node was carried out and confirmed melanoma progression.

**Figure 2:**
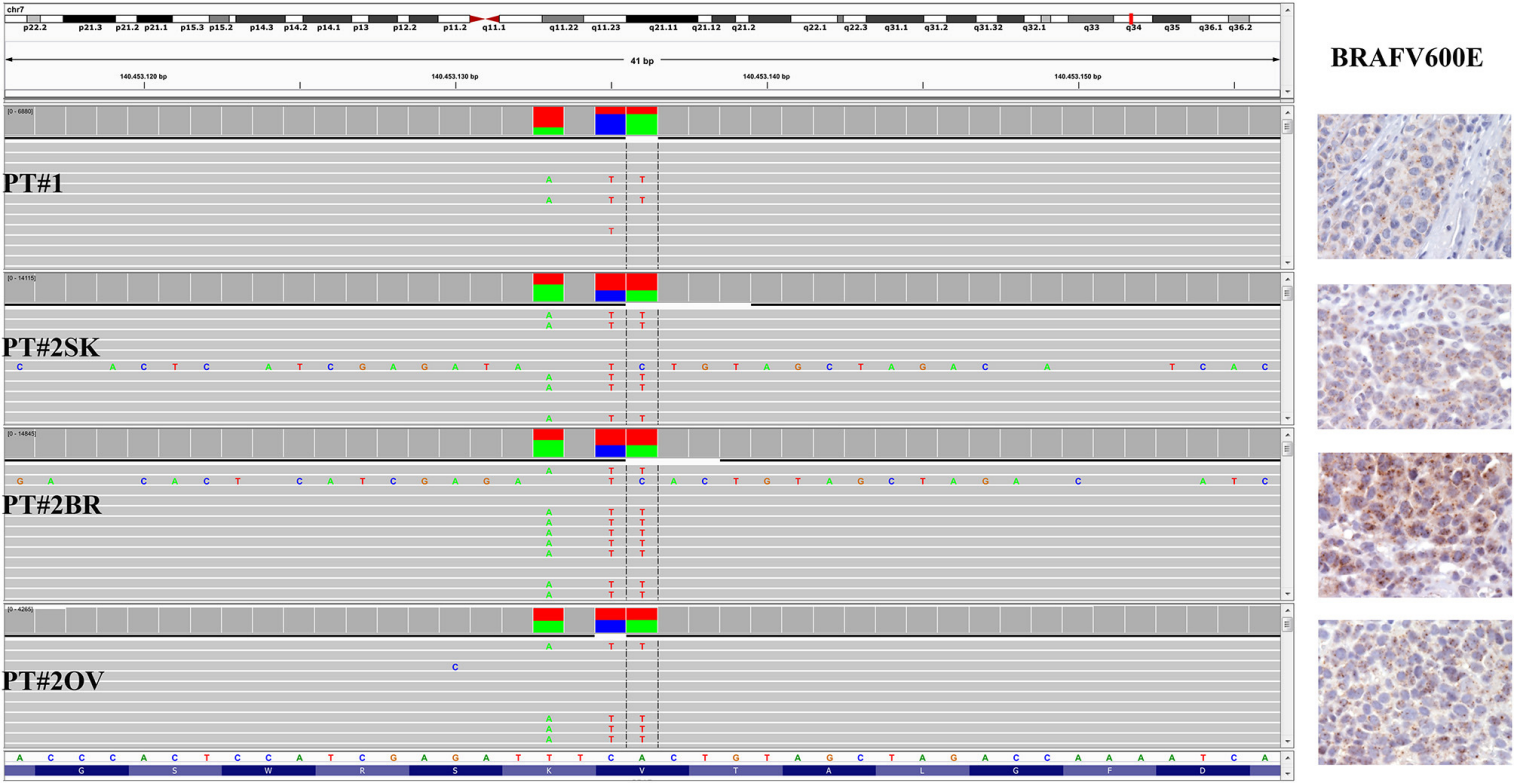
BRAF status in PT#1 and PT#2. Sections are from PT#1 and PT#2 and stained for anti-VE1 as labeled. Primary (PT#1 and PT#2^sk^) and metastatic (PT#2°*v* and PT#2^br^) melanomas are illustrated. A granular cytoplasmic stain for anti-BRAFV600E was detected in melanoma cells from all samples; no reactivity is observed in endothelial cells an immune cells. Sections were counterstained with hematoxylin. Magnification: 400x. Sequencing data illustrate the BRAF substitutions (Igv screenshot of amplicon that cover codon 600 and 601 of BRAF) at codon V600 and K601 in PT#1 and PT#2 by using Ion S5 system (PT#1) and Illumina MiSeq (PT#2^sk^, PT#2^ov^, and PT#2^br^).

**Figure 3:**
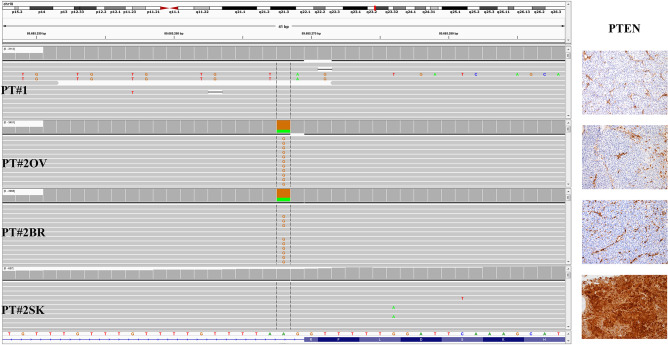
PTEN status in PT#1 and PT#2. Sections are from PT#1 and PT#2 and stained for anti-PTEN as labeled. Primary (PT#1 and PT#2^sk^) and metastatic (PT#2°*v* and PT#2^br^) melanomas are illustrated. Loss of PTEN reactivity was observed in melanoma cells from all samples except PT#2^sk;^ internal positive control are represented by vessel. Sections were counterstained with hematoxylin and imaged at 100x magnification. Sequencing data illustrate the PTEN sequencing analysis (Igv screenshot of amplicon that cover exon 2 of PTEN) by Illumina MiSeq in P#T1 and PT#2 samples. A PTEN substitution is observed in samples PT#2^ov^ and PT#2^br^. sk, skin; ov, ovary; br, brain.

Immunohistochemistry detecting anti-VE1 (antibody recognizing BRAF p.V600E) showed a tiny sparse granular cytoplasmic reactivity (Figure 1). BRAF mutation analysis performed by mass spectrometry and pyrosequencing suggested a complex mutation at position V600 and K601 (not shown), subsequently confirmed by Sanger sequencing (not shown). By using a fifty-six-genes NGS cancer panel (Table S1), detection and confirmation was achieved of a tandem mutation affecting the V600 and K601 codons and showed a three base pair substitution at the genomic level c.[1799_1800delinsAA; c.1802A>T] (Figure 2) from the tissue source. The base pair substitutions were at similar allelic fractions and resulted in cis in term of allele distribution, leading to the p.V600E2; K601I change (Table 1). No other gene abnormalities were detected using NGS, whereas a PTEN loss was detected via immunohistochemistry (Figure 3). The same molecular profile was identified at the primary cutaneous site by Sanger sequencing (not shown). A Cyberknife was performed on all brain metastases, followed by systemic treatment with dabrafenib and trametinib. The patient received dabrafenib at 150 mg BID and trametinib 2 mg QD. No dose variation was carried out during all treatment period. The patient's adherence to target agent combination was accurate and no side effects were documented. A computer tomography (CT) scan performed after 3 months documented a partial response that became progressing to a complete response at the subsequent restaging after 6 months, according to Recist 1.1 criteria (20). Twenty-eight months post treatment initiation, the patient was hospitalized for a cognitive impairment associated with delirium, but brain tumor progression was excluded. A geriatric assessment confirmed the diagnosis of dementia. The treatment was therefore discontinued. The cognitive disorder contraindicated other systemic treatments; antipsychotic, antidepressant, and anxiolytics were used as symptomatic approaches.

**Table 1:** BRAF and PTEN variants identified by the Myriapod® NGS-IL 56G Onco Panel in PT#1 and PT#2.

**Allele variant**	**PT#1**	**PT#2sk**	**PT#2ov**	**PT#2br**
BRAF c.1802A>T	48.72%	24.42%	58.74%	58.71%
	(1,899/1,804)	(2,522/815)	(2,823/4,019)	(6,163/8,764)
BRAF c.1800G>A	49.04%	24.67%	58.79%	58.73%
	(1,879/1,808)	(2,519/825)	(2,817/4,018)	(6,161/8,766)
BRAF c.1799T>A	48.98%	24.54%	58.78%	58.71%
	(1,881/1,806)	(2,519/819)	(2,816/4,016)	(6,161/8,760)
PTEN c.165-2A>G	Absent	Absent	76.1%	71.22%
splice_acceptor_variant&intron_variant			(822/2,618)	(1,154/2,856)

Four months later, liver tumor progression was instrumentally recorded consisting of five metastatic *de novo* lesions with a maximum diameter of 15 mm; no confirming biopsy was performed. The patient was initiated onto a palliative care program. At the last follow-up visit (January 2020, after 38 months from the diagnosis of the metastatic disease and after 12 months from the treatment discontinuation), the patient was still alive and further clinical investigations and assessments showed brain tumor remission and a stable disease at the liver. Timeline with relevant data is included in Figure S1.

### Clinical and Genetic Findings: Patient#2 (Pt#2)

A 26-years-old female presented at clinic with a diagnosis of thick nodular melanoma of the skin trunk (from here referred as Pt#2^sk^) (Breslow thickness of 5.2 mm, ulcerated) (Figure 1), during her first pregnancy. She had no comorbidities, nor past interventions; no familial melanoma was reported. No treatment other than resection of the pigmented lesion was performed, in accordance with the patient's choice. The patient presented after a 5 years gap and was pregnant in her 30 weeks of gestation. At this visit it was decided to carry out a brain MRI, following the presentation in the clinic of acute neurological symptoms. Five brain metastases were detected (SLD 80 mm) with the largest showing a diameter of 40 mm and involving the frontal horn of the left lateral ventricle; supratentorial hydrocephalus was observed in both lateral ventricles. At week 31, a cesarean section combined with right ovariectomy (from here referred as Pt#2°*v*) and a brain (from here referred as Pt#2^br^) symptomatic metastasectomy were carried out (Figure 1). Histological evaluation and examination confirmed melanoma infiltration at all suspected sites. A written informed consent was collected from the patient before all further genetic analyses commenced. The BRAF mutation analysis performed, on genomic DNA extracted from all three melanoma sites using Mass Spectometry and Pyrosequencing, suggested a complex BRAF mutation involving codon V600 and K601 (not shown). Sanger sequencing of the BRAF exon 15 and the NGS-cancer panel confirmed the presence of the same three base pair substitution at the genomic level c.[1799_1800delinsAA; c.1802A>T] found in Pt#1 (Figure 2 and not shown) and leading to the p.V600E2; K601I change. Moreover, the NGS-cancer panel could also identify a pathogenic PTEN substitution (c.165-2A>G) in Pt#2^ov^ and Pt#2^br^ (Figure 3), leading to complete lack of PTEN expression by immunohistochemistry (Figure 3).

After childbirth, a CT scan documented subcutaneous, cerebral, and lung metastases; LDH levels were normal (within acceptable ranges), and PS was 2, despite the assumption of corticosteroids. At the baseline after surgery, the sum of extracranial lesions diameter was 76 mm, while residual intracranial-tumor burden was 60 mm. The patient started the combination therapy with dabrafenib and trametinib. She received dabrafenib at 150 mg BID and trametinib 2 mg QD. No dose variation was carried out, during all treatment period. The patient's adherence to target agent combination was accurate and no side effects were documented. After 12 weeks of therapy, any clinical disease progression was assessed using a further CT scan. Imaging evaluation documented a partial response of extra-cranial disease and a progression of intracranial disease (parenchymal and meningeal), according to Recist 1.1 criteria (20). No other intervention was possible due to the rapid symptomatic worsening. The brain and meningeal disease progression resulted in the death of the patient after 6 months. Timeline with relevant data is included in Figure S2.

Data on the functional activation of the MAPK pathway in these tandem mutations is lacking. We tested the MAPK activation in *Pt#1*^*sk*^ and *Pt#2*^*sk*^ by immunohistochemistry. Of note, melanoma cells from both cases co-stained for anti-phospho-p44/42 MAPK (Thr202/Tyr204) (Figure S3).

## Discussion

BRAF mutants have been categorized into classes based on biochemical and signaling mechanisms. BRAF V600D/E/K/R are referred to as type I BRAF mutations, resulting in RAS-independent active monomers showing marked activation of kinase activity within signaling pathways(s) (3) as well as being sensitive to BRAF and MEK inhibitor combinations. In contrast to this *in vitro* characterization of non-V600 BRAF mutants has identified two additional subgroups including class II mutants with intermediate to high kinase activity and class III mutants that lack or possess low kinase activity. Non-V600 class II mutant function as RAS-independent activated dimers and those affecting the K601 position are capable of expressing high kinase activity, but do not readily respond to BRAF inhibitor in monotherapy (3); moreover, there is a paucity of current published data on the effects of MEK inhibitors alone or in combination with BRAF inhibitors (3, 21). This study reports the genomic and clinical features of two melanoma patients (Pt#1 and Pt#2) showing a rare complex mutation affecting codon V600 and K601 of the BRAF gene, leading to a V600E2; K601I change. This rare mutational event was initially investigated using state-of-the-art tools such as immunohistochemistry for VE-1, mass spectrometry and pyrosequencing, and has been recently further refined using an NGS-based detection approach.

To the authors' knowledge, the tandem mutation V600E2; K601I was first described in a retrospective report, detected by pyrosequencing in a cutaneous melanoma metastasis (22). This genomic abnormality is still not being reported in the most widely accessed catalog of somatic mutations (i.e., COSMIC; ClinVar) and no preclinical or clinical models have explored the effect of a double amino-acid substitution on the BRAF structure and on its kinase activity. The authors believe that the efficacy of modern targeted therapies in patients whose tumors harbor this rare mutation has never been described. This study reports the first experience regarding the efficacy of modern target therapy in two patients bearing BRAF p.V600E2; K601I mutated melanoma. As detailed above, it is easily conceivable that this new mutation maintains the sensitivity to combination treatment as typical for V600E substitution. This hypothesis is in accordance with the initial responses observed in both patients. Recently Menzer et al. explored the therapeutic role of BRAFi/MEKi in patients with metastatic melanomas harboring rare variants of non-V600E/K BRAF mutation (19). Their analysis did not include a cohort of seven patients with a class I mutation (V600E/K) combined with a rare BRAF mutation since the presence of the former was considered to be predictive of response to targeted therapy. In this cohort of seven patients, the overall response rate was about 40%, while PFS value ranged between 0.3 and 43.2 months and OS value between >0.4 and >56.5 months. “Pooled” data analysis of tandem mutations, including the seven patients from Menzer et al. (19) and our two patients, documented a median PFS of 3 months with target therapy, while median OS was not reached (Table S2). The retrospective nature, the limited number of patients included and the lack of detailed prognostic variables (i.e., LDH level, tumor burden, ECOG Performance Status) has limited the ability to fully understand the findings of the analysis. Moreover, no other genomic abnormalities except for BRAF mutation were detected.

In our case series, overall survival and duration of the treatment response differed between the two cases described, mainly as a consequence of the baseline extent of brain involvement and intracranial treatment failure. The cerebral SLD was less extended in PT#1 compared to PT#2, respectively 40 and 80 mm. As recently documented in a phase II COMBI-MB trial, combination treatment has improved intracranial disease control in comparison to historical outcomes for patients treated with loco-regional therapies alone (23). On the other hand, median duration of the overall response to BRAF and MEK inhibitors in patients enrolled in COMBI-MB trial was half than that observed in patients without brain involvement (9, 23). In agreement with the COMBI-MB results, the prognosis of PT#2 was poor, due to symptomatic brain metastases at the baseline, to the short-lived response and to rapid intracranial disease progression. Furthermore, “pooled” analysis data demonstrated that the baseline tumor load is a strong predictor of target therapy response (9, 24, 25) and new CNS lesions at progression after initial scan detection diagnosis have been associated with a poor response and even poorer prognosis (26). The tumor burden is closely related to molecular heterogeneity that could foster the occurrence of acquired resistance and could affect target applied therapy efficacy.

In addition, it should be noted that in both cases investigated in this work PTEN loss at the protein level by immunohistochemistry was detected. PT#1 showed lack of PTEN in the primary. On the contrary, in PT#2, PTEN expression was lost in the metastatic lesions, but preserved at the primary site, suggesting a role in melanoma dissemination. On note, in the metastatic lesions of PT#2 PTEN mutation was also detected using the NGS-based cancer panel. Specifically, we found an intron 2 PTEN c.165-2A>G substitution. The latter, which has been reported at the germline level in patients with Cowden syndrome, leads to the creation of new cryptic splice sites resulting in the frameshift Phe56Valfs^*^7 (27).

PTEN loss represent a molecular driver for melanoma progression (28). As detailed by the comprehensive molecular TGCA analysis, PTEN deletion/mutation was more frequent in BRAF mutated tumors and the co-occurrence of both mutations has been described in 20% of treatment-naïve melanomas (29). Recent findings indicate that this combination (BRAF mutation and PTEN loss) does not preclude response to BRAF and MEK inhibitors, as in the study carried out here. This concurs with previous observations where complete or partial responses to BRAF inhibitor alone were also observed in patients with a loss of PTEN expression, detected on pre-treatment tumor samples (30). Frequent mechanisms involved in BRAF/MEK inhibitors resistance of melanoma converge in the reactivation of the BRAF-MEK-ERK pathway usually following NRAS mutation (31), alterations in BRAF splicing (32), and BRAF amplification (33, 34), activation of IGF1R-ERK5 pathway (35) as well as in the activation of the PI3K-Akt signaling (36). The role of the activation of the compensatory PI3K/AKT signaling cascade in the acquired resistance of melanomas patients to BRAF/MEK inhibitors still remains controversial, although oncogenic PIK3CA and AKT3 mutants enables the survival of a dormant population of MAPK-inhibited melanoma cells. The evolution of resistance in surviving tumor cells was associated with MAPK re-activation and no longer a dependence on the initial PI3K/AKT-activating oncogene. This dynamic form of resistance alters signaling dependence and may lead to the evolution of tumor sub-clones highly resistant to multiple targeted therapies (37). This mechanism could be advocated in favoring brain metastases resistance and progression (38). These findings add to the growing literature supporting the presence of brain metastasis-specific molecular aberrations involving the activation of PI3K/AKT pathway. Furthermore, loss of PTEN has been correlated with accelerated brain progression in patients with stage IIIB/C, supporting the rationale for further evaluation of this mutation and suggesting the importance of PI3K/AKT pathway as a potential therapeutic target (39).

In conclusion, this study of V600E2/K601I tandem mutation, in the authors opinion, shows an increased response when combined BRAF/MEK target therapy is introduced. Indeed, this treatment has proven to be effective and safe in the cases described above. In accordance with previous observations, at progression, both patients shared the same phenotype characterized by the co-occurrence of PTEN loss and BRAF mutation. Intracranial treatment failure in PT#2 could be explained as a consequence of higher tumor burden and molecular heterogeneity. Accordingly, PTEN loss could variably modify the MAPK signaling circuits and modulate the resistance to BRAF/MEK inhibitors.

## Data Availability Statement

All datasets generated for this study are included in the article/Supplementary Material.

## Ethics Statement

Ethical review and approval was not required for the study on human participants in accordance with the local legislation and institutional requirements. The patients/participants provided their written informed consent to participate in this study. Written informed consent was obtained from the individual(s) for the publication of any potentially identifiable images or data included in this article.

## Author Contributions

WV, FC, and AB: conceptualization and supervision. GB, MP, DM, and EG: methodology. EG: validation. DM, MB, BL, CZ, FZ, VT, FC, WV, GB, and MB: formal analysis. DM, MB, BL, CZ, FZ, VT, FC, GB, MB, and EG: data curation. FC, WV, GB, MP, and EG: writing—original draft preparation. FC, WV, and EG: writing—review and editing. All authors have read and agreed to the published version of the manuscript. All authors contributed to the article and approved the submitted version.

## Conflict of Interest

GB and MP were employed by Diatech Pharmacogenetics SRL. The remaining authors declare that the research was conducted in the absence of any commercial or financial relationships that could be construed as a potential conflict of interest.
