# Supplementary material for: A Rare Complex BRAF Mutation Involving Codon V600 and K601 in Primary Cutaneous Melanoma: Case Report
Source: Front Oncol. 2020 Jul 10;10:1056. doi: 10.3389/fonc.2020.01056 (PMC7367153; doi:10.3389/fonc.2020.01056)
Supplement: Supplementary file 1 [file Table_1.DOCX]

**Supplementary Table S1.** The list of 56 cancer related-gene analyzed by the Myriapod^®^ NGS-IL 56G Onco Panel. The panel allows to investigate hot spot mutation and the entire coding region*.

| ABL1 | CSF1R | FBXW7 | GNAS | KIT | NPM1 | SKT11 |
| --- | --- | --- | --- | --- | --- | --- |
| AKT1 | CTNNB1 | FGFR1 | HNF1A | KRAS | NRAS | SMAD4 |
| ALK | DDR2 | FGFR2 | HRAS | MAP2K1 | PDGFRA | SMARCB1 |
| APC | DNMT3A | FGFR3 | IDH1 | MET | PIK3CA | SMO |
| ATM | EGFR | FLT3 | IDH2 | MLH1 | PTEN | SRC |
| BRAF | ERBB2 | FOXL2 | JAK2 | MLP | PTPN11 | TP53* |
| CDH1 | ERBB4 | GNA11 | JAK3 | MSH6 | RB1 | TSC1 |
| CDKN2A | EZH2 | GNAQ | KDR | NOTCH | RET | VHL |

| \| **Braf Genotype** \| **Treatment** \| **N** \| **ORR (n)** \| **MEDIAN PFS Months CI (95%)** \| **MEDIAN OS**  **Months CI (95%)** \| \| --- \| --- \| --- \| --- \| --- \| --- \| \| V600E + V600V (19) \| Brafi+Meki \| 1 \| CR \| PFS = 43.2 \| OS > 56.5 \| \| V600E +V600R (19) \| Brafi+Meki \| 1 \| PD \| PFS = 0.3 \| OS > 0.4 \| \| V600E +V600M (19) \| Brafi+Meki \| 1 \| PR \| PFS= 11.3 \| OS =20.3 \| \| V600K + V600R (19) \| Brafi+Meki \| 1 \| PR \| PFS > 1.9 \| OS > 1.9 \| \| V600E + K601E (19) \| Brafi+Meki \| 1 \| PD \| PFS =1.4 \| OS > 29.3 \| \| V600E + W604C (19) \| Brafi \| 1 \| SD \| PFS > 1.1 \| OS > 1.1 \| \| V600E + F610L (19) \| Brafi+Meki \| 1 \| PD \| PFS = 2.0 \| OS = 2.1 \| \| V600E2+K601I (Consoli et al) \| Brafi+Meki \| 1 \| RC \| PFS=32 \| OS =38 \| \| V600E2+K601I (Consoli et al) \| Brafi+Meki \| 1 \| PD \| PFS=3 \| OS=6 \| \| Pooled analysis \|  \| 9 \|  \| PFS=3 (CI 95% 0-14.3) \| OS not reached \| |  |  |  |  |  |
| --- | --- | --- | --- | --- | --- | --- | --- | --- | --- | --- | --- | --- | --- | --- | --- | --- | --- | --- | --- | --- | --- | --- | --- | --- | --- | --- | --- | --- | --- | --- | --- | --- | --- | --- | --- | --- | --- | --- | --- | --- | --- | --- | --- | --- | --- | --- | --- | --- | --- | --- | --- | --- | --- | --- | --- | --- | --- | --- | --- | --- | --- | --- | --- | --- | --- | --- | --- | --- | --- | --- | --- |

**Table S2.** Pooled analysis of patients harboring a tandem mutation.
