# Supplementary material for: A Rare Complex BRAF Mutation Involving Codon V600 and K601 in Primary Cutaneous Melanoma: Case Report
Source: Front Oncol. 2020 Jul 10;10:1056. doi: 10.3389/fonc.2020.01056 (PMC7367153; doi:10.3389/fonc.2020.01056)
Supplement: Supplementary file 2 [file Data_Sheet_1.docx]

**SUPPLEMENTARY APPENDIX**

**METHODS**

## DNA Extraction and Sequencing

Tumor sample analysis: H&E stained slides were reviewed to ensure sufficient viable tumor content. Total genomic DNA was extracted from five 5 micron-thick unstained sections of FFPE tissue blocks according to manufacturer’s instructions, using an automated DNA Extractor (Maxwell RSC DNA FFPE Kit, Promega). The total DNA was eluted in 100ul and immediately stored at -20°C after spectrophotometry (Nanodrop 2000). BRAF mutation analysis was performed by Pyrosequencing, Mass Spectometry and Sanger Sequencing. Briefly, Pyrosequencing was performed on the PyroMark Q96 ID instrument with commercially available kit (BRAF Status, Diatech Pharmacogenetics, Italy) according to manufacturer’s instructions. Mass spectrometry was performed by MassARRAY system (Sequenom Inc, San Diego, CA, USA) using the CE-IVD Myriapod Colon Status kit (Diatech Pharmacogenetics, Italy). Finally, for Sanger sequencing, exon 15 of the BRAF gene was amplified by polymerase chain reaction (PCR) using the following primers: 5′-TGCTTGCTCTGATAGGAAAATG-3′ (forward) and 5′-AGCATCTCAGGGCCAAAAAT-3′ (reverse). The amplification protocol included denaturation at 94°C for 2 minutes; 40 cycles of denaturation at 95°C for 10 seconds, annealing at 58°C for 30 seconds, and extension at 72°C for 30 seconds; followed by a final extension at 72°C for 7 minutes using Amplitaq Gold polymerase (Life Technologies). PCR products were electrophoresed on 2.5% agarose gels and subsequently sequenced using the same forward and reverse primers and Big Dye Terminator v 3.1 (ABI Systems, Applied Biosystems, Foster City, CA, USA). DNA sequences and the BRAF mutations were determined using an ABI 3130 automatic sequencer (Applied Biosystems).

## NGS Sequencing

Sequencing amplicon libraries were prepared using the commercially available kit Myriapod^®^ NGS-IL 56G Onco panel (Diatech Pharmacogenetics srl; <https://www.diatechpharmacogenetics.com/en/>), according to the manufacturer’s instructions. The panel contains primers to generate 263 amplicons, with a size ranging from 92 to 184 bp, from 56 cancer-related genes (see **Table 1**).  Briefly, libraries were prepared via multiplex PCR single-tube amplification using 10-25ng of DNA according to quantity and the degree of fragmentation calculated with the Real Time PCR reaction included in the kit. PCR libraries were cleaned-up with SPRIselect beads (Beckman Coulter Inc; <https://www.beckman.it/en/reagents/genomic/cleanup-and-size-selection/size-selection>) following manufacturer’s instructions and specific Illumina adapters D7XX and D50X were added through a quick ligation reaction in a unique combination for each sample. Indexed products were enriched by 4-cycles PCR reaction to select and enrich the fragments that were properly indexed. To exclude residual primers and PCR by-products, enriched libraries were cleaned-up with SPRIselect beads following manufacturer’s instructions and finally quantified with Qubit 4.0 Fluorometer using Qubit® dsDNA HS Assay Kit protocol (Thermo Fischer Scientific inc.). The indexed and enriched libraries were then normalized to a concentration of 2 nM, and equal volumes were pooled and diluted with NaOH 0.2 N to generate the final sequencing pool (10pM). The pool was sequenced using a MiSeq instrument with MiSeq Reagent Kit V2 (300 cycles) flow cell (Illumina Inc.), using a paired-end sequencing protocol, according to the manufacturer’s instructions. All the reagents for DNA quantification (Real-Time PCR), PCR multiplex, Indexing and Enrichment steps are included in the Myriapod^®^ NGS-IL 56G Onco panel (Diatech Pharmacogenetics srl).

Bioinformatics analyses were conducted using Myriapod NGS – Data Analysis Software, developed by Diatech Pharmacogenetics srl, with a proprietary analysis pipeline specific for somatic mutations and InDels analysis. Briefly, raw sequenced reads were aligned against the human genome (reference hg19) using BWA. Subsequently, raw reads were trimmed for quality scores, while PCR primers were removed. The resulting BAM files were locally re-aligned, such that the number of mismatching bases (InDels) is minimized across all reads, and then recalibrated to stabilize the reads’ quality value. Variant Calling is performed using a proprietary pipeline based on a machine learning approach (<https://www.diatechpharmacogenetics.com/en/myriapod-ngs-line/>). The variants detected were then filtered (coverage < 500x; VAF < 0,05; StrandBias; Recalibrated Quality < 1000) and annotated with the actual transcript, and using several population DataBases, including dbSNP, 1000 Genomes, Exac, Esp.

## Immunohistochemistry

Immunohistochemistry was performed for BRAFV600E (clone VE, mouse, ready to use, Roche) and PTEN (clone 6H2.1, mouse,1:100, Dako) on primary and metastatic melanoma samples from PT1 and PT2. Four-micron thick tissue sections were cut from formalin-fixed, paraffin-embedded blocks and subsequently de-paraffinized and rehydrated in graded solutions of ethanol and distilled water. Endogenous peroxidase was blocked by incubation with methanol and hydrogen peroxide 0,03% for 20 minutes during rehydration. BRAFV600E reaction was performed on BenchMark Ultra System (Ventana). PTEN (clone 6H2.1, 1:100, Dako) was carried out using microwave oven epitope retrival in ethylenediamine tetra-acetic acid (EDTA) buffer (pH 8.0) and revealed by Novolink Polymer (Leica Microsystems) followed by DAB. Finally, the slides were counterstained with Meyer’s Haematoxylin.
