# Supplementary figures and images for: A Rare Complex BRAF Mutation Involving Codon V600 and K601 in Primary Cutaneous Melanoma: Case Report
Source: Front Oncol. 2020 Jul 10;10:1056. doi: 10.3389/fonc.2020.01056 (PMC7367153; doi:10.3389/fonc.2020.01056)

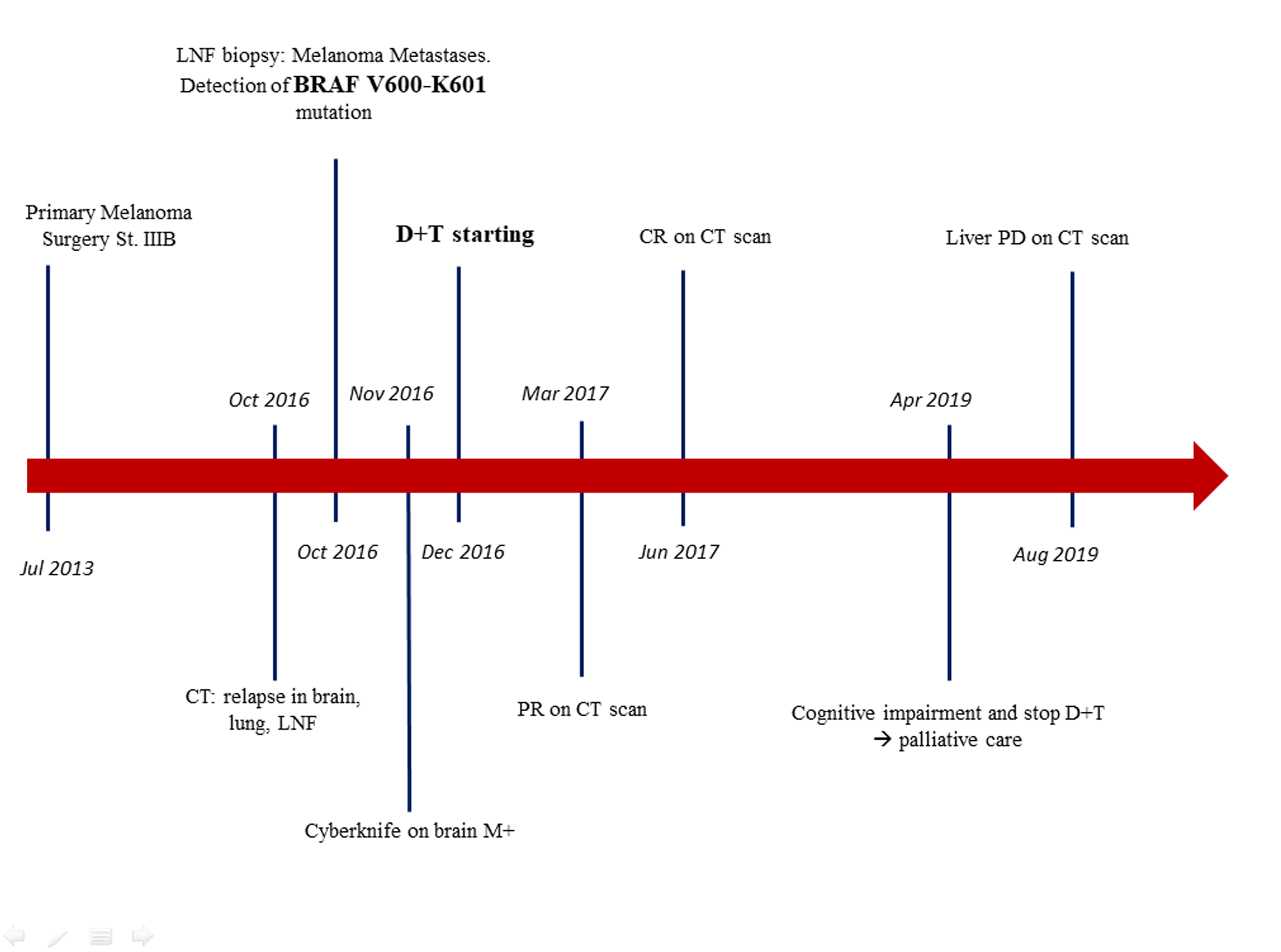


**Figure S1**. Timeline PT1#: description of relevant events, characterizing patient’s history.

Supplement: Supplementary file 3 [file Data_Sheet_2.docx]

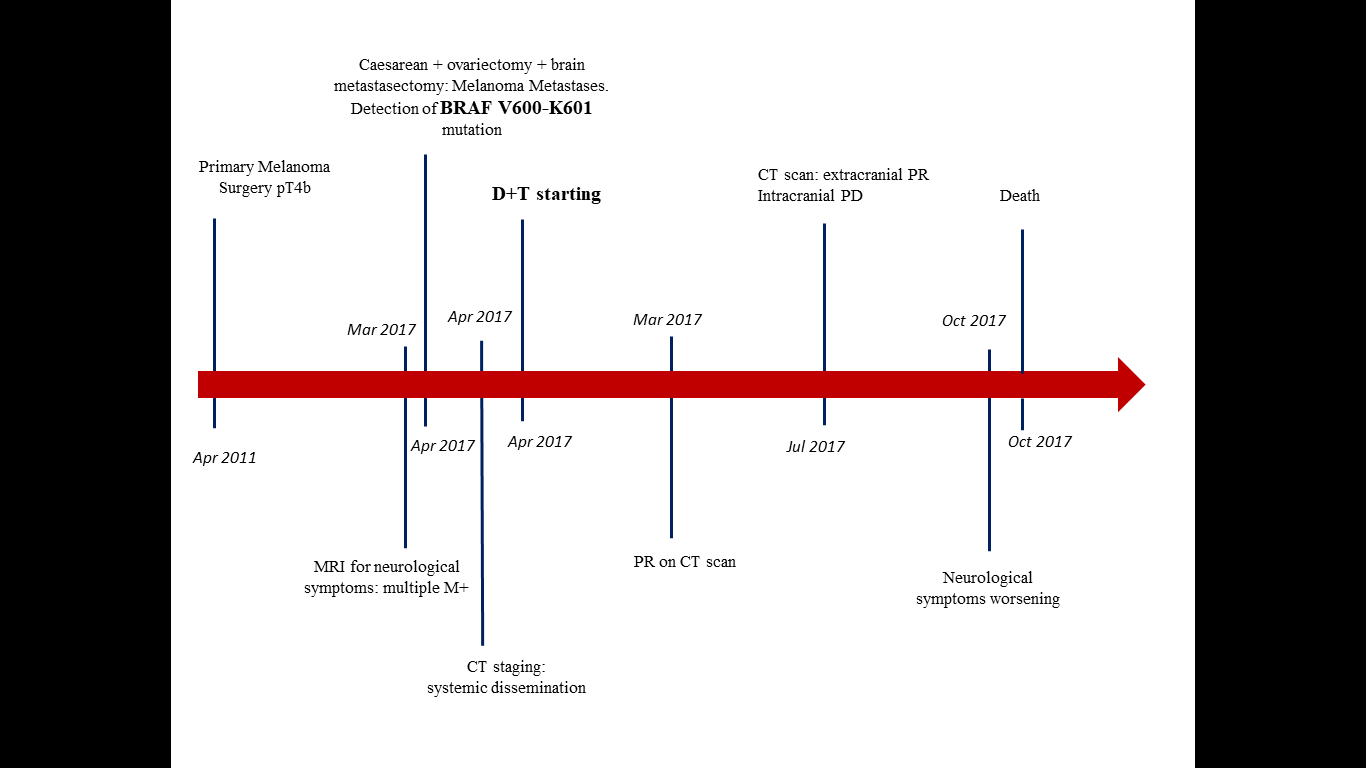


**Figure S2.** Timeline PT2#: description of relevant events, characterizing patient’s history.

Supplement: Supplementary file 4 [file Data_Sheet_3.docx]
