# Supplementary material for: A Rare Complex BRAF Mutation Involving Codon V600 and K601 in Primary Cutaneous Melanoma: Case Report
Source: Front Oncol. 2020 Jul 10;10:1056. doi: 10.3389/fonc.2020.01056 (PMC7367153; doi:10.3389/fonc.2020.01056)
Supplement: Supplementary file 5 [file Data_Sheet_4.docx]

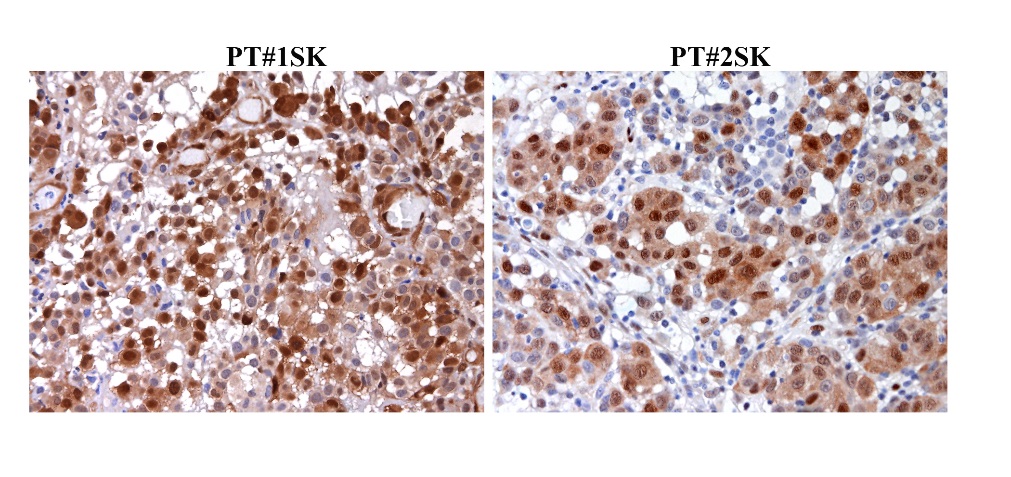


**Figure S3.** Anti-phospho-p44/42 MAPK (Thr202/Tyr204) **in PT#1 and PT#2.** Sections are from PT#1 and PT#2 skin and stained for anti-phospho-p44/42 MAPK. Strong and diffuse phospho-p44/42 MAPK is observed in melanoma cells. Sections were counterstained with hematoxylin. magnification: 200x.
